# Neutrophil-to-lymphocyte ratio for the clinical management of dengue: a systematic review and meta-analysis

**DOI:** 10.3389/fepid.2026.1877646

**Published:** 2026-08-26

**Authors:** Miguel Cabanillas-Lazo, Ivan Alegre-Cordero, Leonardo D. Mera-Lojano, Gerardo Luna-Peralta, Claudia Cruzalegui-Bazan, Patricio Castro-Suárez, Carlos Quispe-Vicuña, Frank Mayta-Tovalino

**Affiliations:** 1 Grupo de Investigación NEMECS: Neurociencias, Metabolismo, Efectividad Clínica y Sanitaria, Universidad Científica del Sur, Lima, Perú; 2 Sociedad Científica de San Fernando, Lima, Perú; 3 Facultad de Medicina de San Fernando, Universidad Nacional Mayor de San Marcos, Lima, Perú; 4 Faculty of Medical Science, Central University of Ecuador, Quito, Ecuador; 5 Vicerrectorado de Investigación, Universidad San Ignacio de Loyola, Lima, Perú

**Keywords:** dengue, lymphocytes, meta-analysis, neutrophils, review

## Abstract

**Introduction:**

Dengue fever (DF) remains a major global public health concern, especially in tropical and low-resource settings. The neutrophil-to-lymphocyte ratio (NLR) has been proposed as an accessible biomarker for diagnosis and severity assessment, but the existing evidence is heterogeneous. To summarize the current evidence on the behavior of the NLR in DF.

**Methods:**

This study conducted a systematic search in five databases for observational studies published until April 2024. Inclusion and exclusion criteria were based on predefined eligibility criteria, and quality and methodological assessment were expressed using the Newcastle–Ottawa Scale. Data was pooled using a random-effects model and results generalized as applicable, when pooling could not be carried out. A summary receiver operating characteristic curve (sROC) was obtained to assess the diagnostic accuracy of NLR for distinguishing DF from COVID-19. The certainty of evidence was graded using the GRADE system.

**Result:**

A total of 6,166 participants from 15 observational studies were included, all with low risk of bias. A single study showed a higher NLR in dengue vs. healthy controls (Bhattarai, 2023; MD: 1.29; 95% CI: 0.87 to 1.71), with moderate certainty. The SROC curve for dengue vs. COVID-19 (2 studies, *n* = 226) showed sensitivity and specificity of 83.9% and an AUC of 0.907, indicating high diagnostic accuracy, with moderate certainty.

**Conclusion:**

For prognosis, severe dengue showed a small, but significant NLR decrease compared with dengue with warning signs (MD: −0.54; 95% CI: −0.91 to −0.17, I2 = 0%), with very low certainty. NLR may serve as a supportive diagnostic biomarker for dengue. However, its prognostic value remains uncertain, highlighting the need for large, prospective studies to validate cutoff points and clinical utility.

## 1 Introduction

Dengue is the most widespread mosquito-borne viral disease, with an estimated 390 million annual infections globally (1, 2), and its incidence continues to rise, with approximately two-fifths of the world's population living in endemic regions (3). Transmitted primarily by Aedes aegypti and Aedes albopictus mosquitoes, the dengue virus (DENV) poses a significant threat in endemic countries, such as those in Asia, including India (4). Understanding the disease's clinical characteristics and laboratory parameters is critical for reducing associated mortality and morbidity (3), particularly given its progression through three distinct pathophysiological stages: febrile, critical, and convalescent phases (2).

The symptoms of dengue are similar to those of other viral infections, and clinical diagnosis based on symptoms and physical examination alone has limited accuracy. This underscores the need for accessible, cost-effective, and reliable diagnostic tools. Currently, the gold standard for diagnosing dengue is virus culture. Other tests, such as genomic detection, antigen detection, antibody detection, or biosensors, are effective tests that detect infection but have the major drawback of cost-effectiveness, specialized equipment, and the need for specialized laboratories (5–8). Rapid diagnostic methods have increased for prognostic evaluation because early detection of severe dengue could guide decision-making. Peptide-based biosensors, graphene-derived nanomaterial sensors, and immuno-magnetic agglutination tests have been evaluated to improve point-of-care detection for predicting complications by quantifying markers such as NS1, cytokines, etc., or specific serotypes (9–11). There is a growing importance of integrating diagnostic and prognostic tools to improve outcomes in dengue infections.

Early dengue infection is strongly associated with leukopenia, generally <5000 cells/mm3, in the critical part of the febrile phase. There is a greater proportional decrease in neutrophils than in lymphocytes, thereby changing the neutrophil-to-lymphocyte ratio (NLR), which could provide a prognosis of disease progression. Leukopenia is followed by a sharp drop in platelets, which often causes plasma leakage (5, 12). The use of NLR as a biomarker of infection is a low-cost and easily measured option and has been correlated with the severity of hemorrhagic manifestations and shock (13–15).

There is evidence that NLR is useful as a biomarker for other diseases, such as neuromyelitis optica spectrum disorder (NMOSD), which is increased in the patient group compared to the healthy control group, and is also associated with poor prognosis (15). NLR independently predicts adverse outcomes in patients hospitalized with community-acquired pneumonia (16), and there has been discussion about its potential as a promising marker for identifying post-surgical infections (17).

At present, there is no consensus on the usefulness of NLR as a biomarker and standardized use for dengue. Based on this background, this systematic review with meta-analysis seeks to summarize the studies conducted to date on the behavior of NLR and perform an in-depth analysis of the association of this marker with dengue.

## 2 Methods

This systematic review and meta-analysis follows the guidelines of the Preferred Reporting Items for Systematic Reviews and Meta-Analyses statement and the Cochrane Handbook for Systematic Reviews of Interventions (18). We have registered our protocol in PROSPERO with the identifier CRD42024538357.

### 2.1 Data sources and searches

A comprehensive systematic search was conducted in the databases PubMed, Embase, Scopus, Web of Science, and Google Scholar for studies published up to April 2024. The search in Google Scholar was specifically designed to identify relevant gray literature. We built the search strategy using both controlled vocabularies (MeSH, Emtree) and free-text terms relevant to “dengue” and “neutrophil-lymphocyte ratio” (Supplementary Material S1-Search strategy) following the Peer Review of Electronic Search Strategies Guidelines (19). We did not impose language or publication date restrictions. No deviations from the registered PROSPERO protocol occurred.

### 2.2 Eligibility criteria

We included studies that satisfied all these conditions: (1) participants with laboratory-confirmed dengue, (2) NLR measured in dengue patients, and (3) analytical observational designs (cohort, case-control, cross-sectional). We excluded narrative reviews, animal studies, case reports, conference abstracts, letters, and editorials. Additionally, we hand-checked reference lists of included studies and relevant reviews for further eligible articles.

### 2.3 Study selection

Two independent reviewers (GLP and CCB) imported search results into reference management software, EndNote X9, removed duplicates, and uploaded the remaining records into the screening tool Rayyan. They screened titles and abstracts independently and in duplicate and, in case of disagreement, resolved conflicts by discussion or with a third reviewer (MCL). Next, they independently and in duplicate reviewed full texts of selected articles and again reconciled any discrepancies. The complete list of all excluded articles is reported in (Supplementary Material S2- Excluded studies).

### 2.4 Outcomes

We aimed to evaluate NLR as a biomarker in patients with dengue across four primary outcomes: (1) DF vs. healthy controls; (2) DF vs. fever of unknown etiology (FUE), COVID-19, malaria, or scrub typhus; (3) classification of DF according to the World Health Organization (cases without warning signs, cases with warning signs and severe dengue) (20); and (4) mortality in dengue. A secondary outcome included its association with hemoglobin levels.

### 2.5 Data extraction

Pairs of reviewers (CCB and GLP) independently and in duplicate extracted data using a standardized form and adjudicated disagreements by consensus or via a third reviewer (MCL). Extracted information included study title, first author, year, design, country, main inclusion criteria, sample size for patients with dengue and controls, age, sex, timing of blood sampling, mean NLR with standard deviation according to sample stratification, sensitivity, specificity, outcomes, and time of follow-up for outcome measure. If data remained unclear or missing, we contacted the corresponding authors by email.

### 2.6 Risk of bias

The quality assessment of the included nonrandomized studies was performed independently by two authors (CQP and GLP) using the Newcastle–Ottawa scale (NOS) (21), assessing the domains of selection, comparability and ascertainment of exposure/outcome. A third reviewer (MCL) functioned as an adjudicator for any discrepancies in scores. Reviewers were blind to the study ascribed star-based score within the categories of each domain. Studies were classified as low risk of bias if they scored ≥6 stars, moderate risk if they scored 4-5 stars, and high risk if they scored <4 stars, applying the same absolute threshold across case-control and cohort studies (maximum 9 stars) and cross-sectional studies (maximum 8 stars, using the NOS adaptation for cross-sectional designs).

### 2.7 Data analysis

We performed meta-analyses using the R statistical package (version 4.3.2) with the metacont function of the meta package (version 7.0-0), using a random-effects inverse variance model to pool mean differences (MD) with 95% confidence intervals (CI). We estimated heterogeneity via I^2^ (low: < 30%; moderate: 30%–60%; high: > 60%) and computed between-study variance (*τ*^2^) using the DerSimonian–Laird estimator. For diagnostic accuracy, we synthesized sensitivity and specificity using a bivariate random-effects model to derive summary ROC curves using the diagmeta package (version 0.5-1) while accommodating heterogeneity in cut-off points. Sensitivity analyses included leave-one-out. For studies reporting median and interquartile range (IQR), values were converted to mean and standard deviation using the method of Wan et al. (22).

### 2.8 Certainty of evidence

Main author (MCL) applied the Grading of Recommendation, Assessment, Development, and Evaluation (23) framework to rate certainty in our results, judging study limitations (risk of bias), imprecision (confidence interval width and sample size), indirectness (generalizability), inconsistency (heterogeneity across studies), and publication bias. We also considered effects of large magnitude, dose-response gradients, and plausible residual confounding under the guidelines of the GRADE handbook (24). Based on this framework, we categorized overall evidence certainty as high, moderate, low, or very low.

For diagnostic test accuracy outcomes (sensitivity, specificity, and AUC), certainty of evidence started at high, following standard GRADE guidance for diagnostic test accuracy studies. All other outcomes (mean differences, odds ratios, and beta coefficients) started at low, consistent with GRADE guidance for observational studies of association.

## 3 Results

### 3.1 Study selection

The systematic search retrieved 193 records. After removing duplicates, 78 studies remained for title and abstract screening, resulting in the inclusion of 15 eligible articles in the final analysis (3, 8, 14, 25–36) (Supplementary Material S3- PRISMA flowchart of included studies).

### 3.2 Characteristics of studies

We included 15 observational studies: 2 case–control, 2 cohort, and 11 cross-sectional, published from 2017 to 2024. Most studies were from Asia and Latin America, with a pooled sample of 6166 subjects. Participant age ranged from 7.6 ± 4.8 years in pediatrics to a median of 60 years in adults. NLR was reported as mean ± SD or median (IQR) across studies. Dengue infection was diagnosed by NS1 antigen, IgM/IgG serology, or WHO criteria. NLR was measured at admission or in the first few days of hospitalization. Full details are provided in Table 1.

**Table 1:** Characteristics of the included studies (*n* = 15).

Study-ID	Country	Study Design	Main inclusion criteria	Age (Mean ± SD)	N dengue (%)	N control (%)	Blood sample time	Outcomes	Follow-up
Agrawal et al. (3)	India	Cross sectional	Age: >18 years Diagnosis: Positive for nonstructural protein 1 (NS1) antigen or IgM antibody positive	29.85 ± 13.40	540 (100%)	NR	At admission	Mortality	At discharge
Ashma et al. (14)	Indonesia	Cross sectional	Age: >18 years Diagnosis: Positive IgM serology for dengue virus and meeting WHO criteria for DHF: - Thrombocytopenia (<100,000 cells/mm^3^) - Hemoconcentration (≥20% rise in hematocrit from baseline)	Median: 25.50 (range: 18–58)	70 (100%)	NR	At admission	- Hemoconcentration	At discharge
Athira et al. (25)	India	Cross sectional	Age: <18 years Diagnosis: Positive IgM/IgG serology with ELISA for NS1 antigen and meeting WHO 2009 criteria for dengue classification: - Thrombocytopenia (<100,000 cells/mm^3^) - Hemoconcentration (≥20% rise in hematocrit from baseline)	7.6 ± 4.8	34 (16.1%)	177 (83.9%)	At admission	Severity classification during hospitalization (WHO 2009)	At discharge
Bansal et al. (26)	India	Cross Sectional	- COVID-19: Positive Rapid Antigen Test (RAT) or RT-PCR. - Dengue: Positive NS1 Antigen Test (platelet count <80,000/*μ*L triggered testing).	NR	18 (8.5%)	Covid-19: 118 Febrile Controls: 71	At admission	Differentiation with other fever	At discharge
Bhattarai et al. (27)	Nepal	Cross sectional	Positive dengue profile test (NS1 and/or IgM-only positive) Fever and body pain	Median: 33 (IQR: 23–50)	177 (50.9%)	Dengue-negative: 171 (49.1%)	NR	Differentiation with healthy controls	At discharge
Chatterjee et al. (28)	India	Prospective cohort	Age: <12 years Positive serology for dengue, scrub typhus, or typhoid fever (via RDT or ELISA)	8.15 ± 2.75	39 (34.5%)	44 (38.9%)	NR	Differentiation with scrub typhus	At discharge
Hasanah et al. (8)	Indonesia	Cross sectional	Age: >18 years Diagnosed with Dengue Hemorrhagic Fever (DHF) or COVID-19.	DHF: Median age = 29 years (range: 18–79). COVID-19: Median age = 51 years (range: 20–80).	95 (17.1%)	COVID-19: 459 (82.9%)	At admission	Severity of dengue fever and differentiation with COVID-19	At discharge
Juliansen et al. (29)	Indonesia	Cross sectional	Age: <18 years Confirmed dengue fever or dengue hemorrhagic fever, based on WHO 2009 guidelines	Median: 10.92 (range: 0.04–18)	527 (66.3%)	268 (33.7%)	NR	Differentiation with other fever	At discharge
Kotepui et al. (30)	Thailand	Case Control	Patients infected only with dengue fever or only with malaria, confirmed by laboratory diagnosis (NS1/IgM/IgG for dengue; Giemsa-stained blood films for malaria).	Median:19 (range: 10–31)	347 (50.80%)	336 (49.20%)	NR	Differentiation with malaria	At discharge
Navyaet al. (31)	India	cross-sectional	Age: >18 years Fever and confirmed dengue infection (positive NS1Ag/IgG/IgM antibody)	NR	99 (100%)	NR	NR	Differentiation with other fever	At discharge
Osuna-Ramoset al. (32)	México	cross-sectional	Age: >18 years Diagnosis of dengue with 2009 WHO dengue guideline Positive nucleic acid test for SARS-CoV-2	Median: 40 (IQR: 27.2)	183 (63.54%)	NR	At admission (between 2nd and 7th day after symptom onset)	Difference between COVID-19 and Dengue	At discharge
Recker et al. (33)	Vietnam	retrospective cohort	Diagnosis with rapid dengue test measuring NS1-IgM/IgG	38.7	1593 (100%)	NR	From the first until the eight-day post admission	Severity	At discharge
Rosso et al. (34)	Colombia	cross-sectional	Age: >15 years Seropositive for NS1 or IgM and confirmed seroconversion	Dengue: median 19.5 years (IQR 15.5–29) COVID-19: median 44 years (IQR 34–57)	32 (74.4%)	11 (25.6%)	At admission	Differentiation with COVID-19	At discharge
Rosso et al. (35)	Colombia	cross-sectional	Age: >14 years Fever or other symptoms (e.g., myalgia, headache, rash) for <10 days. Dengue group: Confirmed by NS1 antigen or IgM seroconversion.	Dengue patients: Median age 25 years (IQR: 17–42). COVID-19 patients: Median age 45 years (IQR: 32–62).	70 (75.3%)	23 (24.7%)	At admission	Differentiation with COVID-19	At discharge
Tran et al. (36)	Vietnam	case-control	Age: >16 years For dengue: NS1-positive test result For scrub typhus (ST): febrile illness (≥37.5°C) + at least one clinical sign (e.g., eschar, rash, lymphadenopathy, myalgia, etc.), and negative tests for malaria/dengue/other infections	Dengue: 20 (10–31) Scrub typhus: 33 (22–45)	387 (63.65%)	221 (36.3%)	At admission	Differentiation with scrub typhus	At discharge

### 3.3 Risk of bias assessment

The methodological quality of the 15 included studies was evaluated using NOS. Of these, two were case-control, two cohorts, and 11 cross-sectional studies. All case-control and cohort studies were rated having a low risk of bias, showing adequate ascertainment of exposure, although some lacked detailed reporting on control selection or follow-up adequacy. Among the cross-sectional studies, all demonstrated a low risk of bias with minor limitations regarding sample size justification, representativeness, or confounder control. Overall, the studies exhibited acceptable methodological quality, supporting the reliability of exposure and outcome assessments across the included evidence [Supplementary Material S4- The Newcastle-Ottawa Scale (NOS)].

### 3.4 DF vs. healthy controls

Bhattarai et al. (27) found that dengue patients had higher NLR than healthy controls, with an MD of 1.29 (95% CI: 0.87 to 1.71) (Table 2).

**Table 2:** Narrative synthesis of the included studies evaluating the neutrophil to lymphocyte ratio (NLR) in patients with dengue fever (DF).

Outcome	Study-ID	Number of analized patients	NLR mean (SD) in case or poor outcome	NLR mean (SD) in control or good outcome	NLR mean difference (CI)	Correlation NLR	NLR cutoff	Effect size	Sensitivity (%)	Specificity (%)	Area under the curve (AUC)	Follow-up
Differential diagnosis												
DF and Malaria	Kotepui et al. (30)	683	1.39 (0.51)	4.45 (3.77)	–3.06 (95% CI: –3.47 to –2.65)	NR	2.8	c*β*: 0.995	71.5% (66.4%; 76.2%)	64.9% (59.5%; 70.0%)	NR	NR
DF and Healthy controls	Bhattarai et al. (27)	348	3.65 (2.73)	2.36 (0.83)	1.29 (95% CI: 0.87 to 1.71)	NR	NR	NR	NR	NR	NR	NR
DF and Scrub typhus	Chatterjee et al. (28)	69	NR	NR	NR	NR	2.0	RPR: 1.11 (95% CI: 0.27–4.58)	NR	NR	NR	NR
DF and Scrub typhus	Tran et al. (36)	608	3.07 (2.68)	2.63 (1.64)	0.44 (95% CI: 0.10 to 0.78)	NR	NR	aOR 0.68 (95% CI: 0.57–0.81)*	NR	NR	NR	NR
Severity												
Hemoglobin levels	Ashma et al. (14)	70	NR	NR	NR	0,314*	NR	aβ: 1.782*	NR	NR	NR	NR
Mortality												
Mortality	Agrawal et al. (3)	540	0.66 (0.26)	0.33 (0.28)	0.33 (95% CI: 0.10 to 0.56)	NR	NR	NR	60.0	91.2	0.909	NR

Mean differences (MD) are calculated as dengue mean minus comparator mean (or poor-outcome mean minus good-outcome mean for the mortality comparison); positive values indicate higher NLR in dengue (or in the poor-outcome group).

SD, standard deviation; CI, confidence interval; NR, not reported; cβ, crude beta coefficient; RPR, relative probability ratio, aOR, adjusted odds ratio; aβ, adjusted beta coefficient.

*p < 0.05.

### 3.5 DF vs. FUE

**Figure 1:**
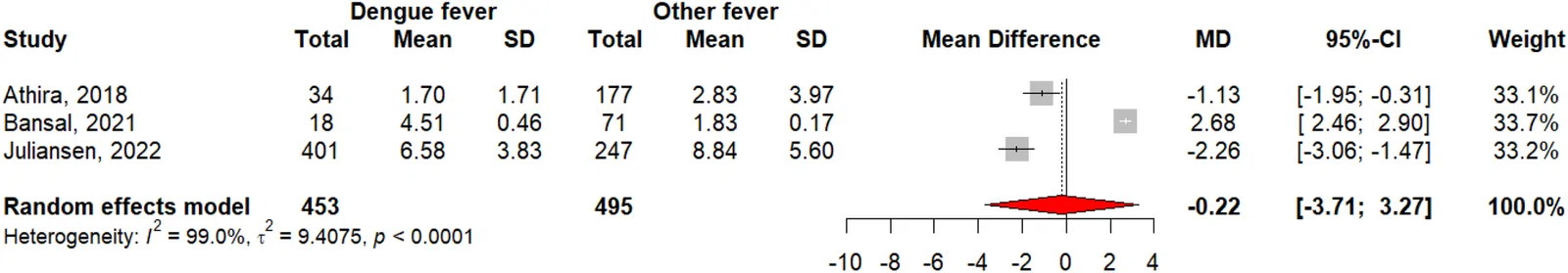
Mean difference in the neutrophil to lymphocyte ratio between patients with dengue fever (DF) and fever of unknown etiology.

Three studies and 948 participants (453 in the dengue group and 495 in the other febrile illness group) were analyzed. The overall analysis showed a non-significant difference in NLR between groups (MD: −0.22; 95% CI: −3.71 to 3.27, I^2^ = 99.0%) (Figure 1). Sequential exclusion of individual studies did not resolve the extreme heterogeneity, with I^2^ remaining near 99% throughout (Supplementary Material S5).

### 3.6 DF vs. COVID-19

Five studies, including 1,114 participants (398 with DF and 716 with COVID-19), were analyzed. Subgroup analysis by geographic region revealed a significant decrease in NLR with substantial heterogeneity (MD: −3.13; 95% CI: −5.99 to −0.27; I^2^ = 99%) (Supplementary Material S6). Sequential exclusion of individual studies did not substantially reduce heterogeneity (Supplementary Material S7).

**Figure 2:**
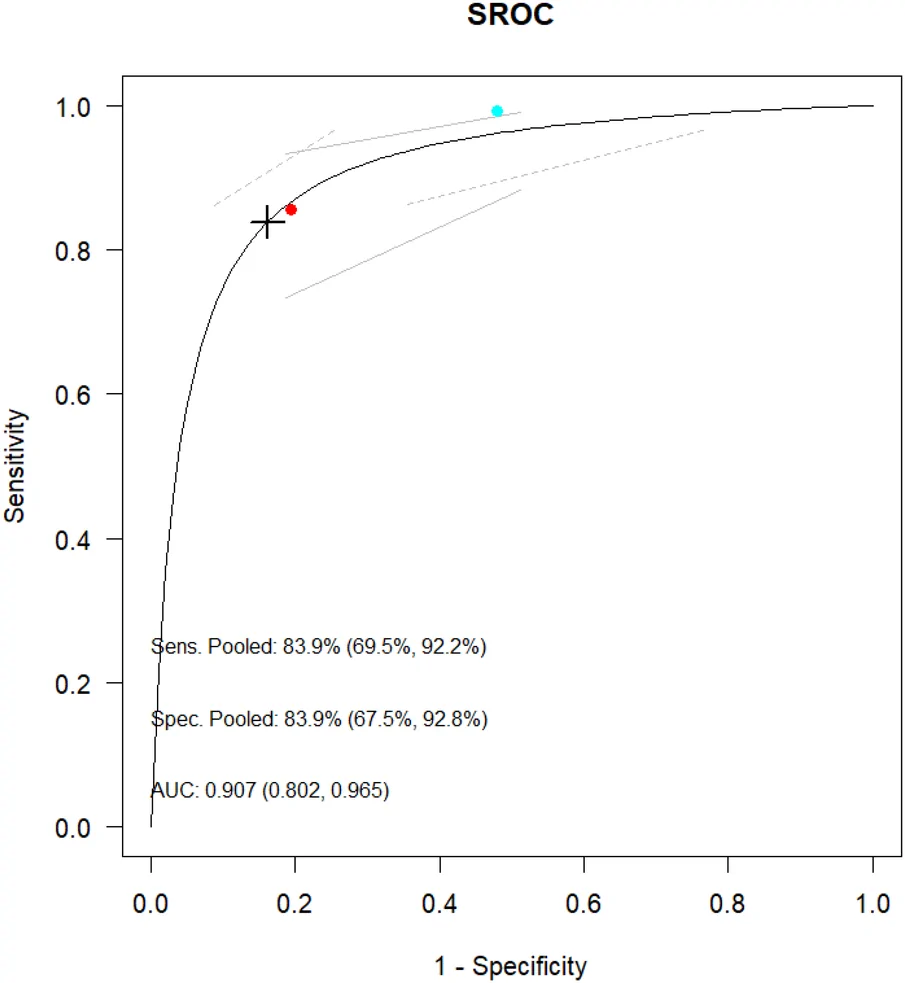
Summary of receiver operating curve of neutrophil to lymphocyte ratio in studies comparing patients with dengue fever and COVID-19 (*n* = 2).

The summary of receiver operating characteristic (SROC) curve showed a sensitivity pool of 83.9% (95% CI: 69.5–92.2%) and a specificity of 83.9% (95% CI: 67.5–92.8%) (Figure 2). The area under the curve (AUC) was 0.907 (95% CI: 0.802–0.965), reflecting high overall diagnostic accuracy in distinguishing DF from COVID-19 infection.

### 3.7 DF vs. malaria

Kotepui et al. (30) evaluated NLR in patients with dengue vs. malaria and reported a significantly lower NLR in dengue compared with malaria, with an MD of −3.06 (95% CI: −3.47 to −2.65), a sensitivity of 71.5%, and a specificity of 64.9% (Table 2).

### 3.8 DF vs. scrub typhus

While Tran et al. (36) observed a higher mean NLR in dengue vs. scrub typhus, MD 0.44 (95% CI: 0.10 to 0.78), and an adjusted OR of 0.68 (95% CI: 0.57-0.81) (Table 2). Similarly, Chatterjee et al. (28) reported a relative percentage ratio (RPR) of 1.11 (95% CI: 0.27-4.58) for the same comparison, though this estimate was imprecise given the small sample size (*n* = 69).

### 3.9 DF severity

A total of three analyses explored NLR according to dengue severity. For DF with and without warning signs, three studies, including 541 patients (111 with warning signs and 430 without) showed a non-significant decrease in NLR with high heterogeneity (MD: −1.51; 95% CI: −3.43 to 0.41; I^2^ = 94%) (Supplementary Material S8). Sequential exclusion of individual studies did not resolve the heterogeneity (Supplementary Material S9).

For severe DF vs. DF with warning signs, three studies, including 135 patients (24 severe and 111 with warning signs), showed a significant decrease in NLR with low heterogeneity (MD: −0.54; 95% CI: −0.91 to −0.17; I^2^ = 0%) (Supplementary Material S10). Leave-one-out analysis confirmed stable pooled effects (Supplementary Material S11).

Similarly, for severe DF vs. DF without warning signs, three studies with 454 patients (24 severe and 430 without warning signs) revealed a non-significant NLR decrease with high heterogeneity (MD: −1.60; 95% CI: −3.65 to 0.45; I^2^ = 87%), and sensitivity analyses confirmed the stability of the results (Supplementary Materials S12, S13).

### 3.10 Hemoglobin levels

Ashma et al. (14) observed an association between NLR and hemoglobin levels (adjusted *β*=1.782, *p* < 0.05; correlation 0.314) (Table 2).

### 3.11 Mortality

Agrawal et al. (3) reported that deceased patients had higher NLR compared with survivors, MD of 0.33 (95% CI: 0.10 to 0.56), with 60% sensitivity, 91.2% specificity, and an AUC of 0.909 (Table 2).

### 3.12 Certainty of evidence

Certainty of evidence started at high for diagnostic test accuracy outcomes (sensitivity, specificity, AUC) and at low for all other observational associations (mean differences, odds ratios, beta coefficients), following standard GRADE guidance. Certainty was subsequently downgraded for most outcomes due to imprecision (wide or unreported confidence intervals and fewer than 300 participants) and inconsistency (high heterogeneity across studies), and upgraded for one outcome due to a large effect size with a narrow confidence interval. Overall, the certainty of evidence ranged from moderate to very low, reflecting both methodological limitations and variability among the included studies (Table 3). This is consistent with GRADE guidance, in which certainty of evidence additionally reflects inconsistency and imprecision at the outcome level beyond individual-study risk of bias.

**Table 3:** Grade summary of findings.

Outcome	№ of participants (studies)	Certainty of the evidence (GRADE)	Anticipated absolute effects	
			Effect size	95% CI
Diagnostic				
DF and healthy controls	348 (1 observational study)	⊕⊕⊕◯ Moderate^a^	MD: 1.29 (0.87; 1.71)	
DF and FUE	948 (3 observational study)	⊕◯◯◯ Very low^b^^,^^c^	MD: –0.22 (–3.71; 3.27)	
Sensibility for DF and COVID-19	226 (2 observational study)	⊕⊕⊕◯ Moderate^d^	83.9% (69.5%; 92.2%)	
Specificity for DF and COVID-19	226 (2 observational studies)	⊕⊕⊕◯ Moderate^d^	83.9% (67.5%; 92.8%)	
Sensibility for DF and malaria	683 (1 observational study)	⊕⊕⊕◯ Moderate^d^	71.5% (66.4%; 76.2%)	
Specificity for DF and malaria	683 (1 observational study)	⊕⊕⊕◯ Moderate^d^	64.9% (59.5%; 70.0%)	
DF and Scrub typhus	608 (1 observational study)	⊕◯◯◯ Very low^d^	aOR: 0.68 (0.57; 0.81)	
Prognostic				
Dengue fever with and without warning signs	541 (3 observational studies)	⊕◯◯◯ Very low^b^^,^^c^	MD: –1.51 (–3.43; 0.41)	
Severe dengue fever and dengue fever with warning signs	135 (3 observational studies)	⊕◯◯◯ Very low^e^	MD: –0.54 (–0.91; −0.17)	
Severe dengue fever and dengue without warning signs	454 (3 observational studies)	⊕◯◯◯ Very low^b^^,^^c^	MD: –1.60 (–3.65; 0.45)	
Hemoglobin levels	70 (1 observational study)	⊕◯◯◯ Very low^e^^,^^f^	aβ: 1.782	
Sensibility for Mortality	540 (1 observational study)	⊕⊕⊕◯ Moderate^f^	60.0%	
Specificity for Mortality	540 (1 observational study)	⊕⊕⊕◯ Moderate^f^	91.2%	

CI, confidence interval; DF, dengue fever; FUE, fever of unknown etiology; MD, mean difference; aOR, adjusted odds ratio.

GRADE Working Group grades of evidence.High certainty: we are very confident that the true effect lies close to that of the estimate of the effect.Moderate certainty: we are moderately confident in the effect estimate: the true effect is likely to be close to the estimate of the effect, but there is a possibility that it is substantially different.Low certainty: our confidence in the effect estimate is limited: the true effect may be substantially different from the estimate of the effect.Very low certainty: we have very little confidence in the effect estimate: the true effect is likely to be substantially different from the estimate of effect.

a Magnitude of effect: Large effect with a narrow 95% CI. Therefore, we upgraded the evidence by one level.

b Inconsistency: High heterogeneity between studies was detected in the meta-analysis. Therefore, we downgraded the evidence by one level.

c Imprecision: The 95% CI crosses the line of no effect. Therefore, we downgraded the evidence by one level.

d Imprecision: Wide 95% CI. Therefore, we downgraded the evidence by one level.

e Imprecision: Fewer than 300 events leads to uncertainty in the effect estimate. Therefore, we downgraded the evidence by one level.

f Imprecision: Confidence interval not reported, likely wide. Therefore, we downgraded the evidence by one level.

## 4 Discussion

### 4.1 Summary

We found that NLR is generally higher in dengue patients than in healthy cohorts, but not different compared to other febrile illnesses, so that it does not have standalone diagnostic value. In contrast, NLR was lower in dengue than in COVID-19 and lower than in malaria, but higher than in scrub typhus, denoting a variable neutrophilic pattern across comparator infections. No difference was found between mild and severe forms according to severity, although some studies found a prognostic role in relation to hemoconcentration and to mortality. Overall, the evidence found is limited and heterogeneous.

### 4.2 NLR as a diagnostic factor

In our meta-analysis, the NLR was higher in dengue than in healthy subjects, but it did not reliably discriminate against other undifferentiated fevers; however, it was lower in dengue than in COVID-19 and malaria, and higher than in scrub typhus. This suggests that NLR has limited usefulness as a single diagnostic marker but may be useful in differentiating dengue infections from infections that present with marked neutrophilia (such as COVID-19 or malaria). These results are consistent with the previous review by Thach et al. (37) that evaluated the potential of various markers to differentiate severe from non-severe dengue. Although this review did not find that inflammatory markers such as neutrophils or lymphocytes had any statistically significant utility, it did indicate significance in platelet count [standardized mean difference (SMD) −0.65; 95% CI: −0.97 to −0.32] and aspartate aminotransferase level (SMD 0.87; 95% CI: 0.36 to 1.38) in discriminating severe from non-severe cases. On the other hand, Fernández et al. (38) reaffirm the idea that combined hematological parameters (rather than a single ratio) tend to offer better discrimination when developing a multivariate model to differentiate dengue from other acute fevers that integrate clinical and hematological findings (such as leukocytes) and report an AUC of 0.65 for distinguishing dengue in their cohort, suggesting that diagnostic utility lies more in combined panels than in NLR alone. Physiopathologically, this is explained by the fact that dengue usually presents with leukopenia and reactive lymphocytic changes in the early stages (39), while infections such as COVID-19 (40) or malaria (41) cause sustained lymphopenia and marked neutrophilia, raising the NLR. Therefore, this marker would work better as a complementary (e.g., NS1, IgM/IgG, or CRP) marker to identify infections with neutrophilic predominance and as a dynamic indicator of inflammation, but its value as a single diagnostic test for dengue in settings with multiple febrile etiologies is limited.

### 4.3 NLR as a severity factor

In our meta-analysis, dengue severity showed a mixed pattern with NLR: comparisons between patients with and without warning signs, and between severe and non-severe forms, were not significant, with wide confidence intervals and high heterogeneity. However, the comparison between severe dengue and dengue with warning signs showed a small but statistically significant decrease in NLR with low heterogeneity, suggesting this specific contrast may carry some prognostic signal, while the broader severity comparisons remain inconclusive. This lack of significance is reflected in some studies in the literature: for example, Böer et al. (42) analyzed a cohort and found that although a monocyte-to-lymphocyte ratio > 0.13 was associated with the severity of dengue hemorrhagic fever, the NLR did not show a strong association on its own. On the other hand, Sadgir et al. (43) reported that patients with NLR < 2 had a higher probability of more severe disease, suggesting that a decrease in NLR could correspond to a particular immune response in severe cases. In addition, another study indicated that elevated NLR values on days 4–6 of the illness were associated with severe dengue, reinforcing the possibility that NLR dynamics throughout the course of the disease have prognostic value (44). Physiopathologically, these findings are supported by the fact that dengue is associated with relative neutropenia and reactive lymphocytosis in the critical phase (45), decreasing the NLR in patients who will progress to capillary leak syndrome, while in the late stages, systemic inflammation and metabolic stress can induce secondary neutrophilia (46), raising the NLR again in those who develop shock or multiple organ failure. Thus, both our results and external evidence suggest that NLR has prognostic value, but its interpretation must be closely linked to the clinical stage of the disease, which explains the variability and heterogeneity observed between studies.

### 4.4 Recommendations for future research

The marked heterogeneity presented in almost all of our outcomes is consistent with a previous review that also highlighted notable heterogeneity in cut-off values, the timing of NLR measurement, and the reference standards used in various studies to evaluate the usefulness of NLR in relation to dengue (47). In general, this heterogeneity in our results may be due to various causes, such as inconsistent case classifications and low statistical power in some studies, as reported in another review (37). Other reasons could be attributed to differences in populations (adults vs. pediatrics) and in the phase of the disease at which the measurement was taken. Certainty of evidence ranged from moderate to very low across diagnostic and prognostic outcomes, driven mainly by inconsistency and imprecision in the effect measures. All of this would hinder the direct extrapolation of NLR values to all clinical contexts and emphasize that our results should be interpreted with caution. These limitations underscore the need for future studies to standardize NLR cut-off points, define the timing of measurement in relation to the clinical phase of dengue, and use prospective designs with adequate samples to properly adjust for confounding factors such as co-infections, baseline haematological values and comorbidities. It would also be desirable for future studies to evaluate how the diagnostic or prognostic performance of NLR changes when combined with other emerging biomarkers (e.g., platelet count, hemoconcentration indices, or immunological markers) in multivariate models. Given the extreme heterogeneity observed in most pooled analyses (I^2^ 87%–99%), pooled point estimates for these outcomes should be interpreted as indicative rather than definitive and should not serve as a basis for clinical decision-making in isolation.

### 4.5 Clinical applicability

From a clinical and public health perspective, our findings suggest that NLR could be integrated as an auxiliary tool within diagnostic and triage algorithms in resource-limited settings where access to confirmatory tests such as NS1, IgM/IgG, or CRP is insufficient. This is consistent with the guidelines of the Integrated Surveillance Strategy, which promotes the use of locally available basic tests (such as routine blood counts) for the initial assessment of febrile illnesses in settings with limited diagnostic resources (48), as well as the international guidelines of the Integrated Management of Childhood Illness (49) and the WHO essential diagnostics list (1), which recommend the use of basic hematological tests in the initial assessment of febrile illnesses in settings with a high endemic burden. Similarly, the APSED III strategy highlights the operational value of accessible hematological indices to strengthen syndromic surveillance in Asia-Pacific (50). Although the low or very low certainty of several of our outcomes limits the use of NLR as an isolated decision-making tool, its low cost, immediate availability, and relative usefulness in certain differential diagnoses support its potential incorporation into health policies aimed at optimizing initial triage and improving early detection in contexts where confirmatory tests are not widely available.

### 4.6 Limitations and strengths

This systematic review has some limitations. First, most studies were cross-sectional and retrospective, which may limit the ability to establish a clear causality between the effect of NLR and outcomes; furthermore, these results could also be subject to unmeasured confounding factors. Second, several sources of clinical and methodological heterogeneity likely explain the extremely high I^2^ values observed across most comparisons (87%–99%): NLR cut-off values varied substantially between studies with no consensus threshold; the timing of blood sampling relative to symptom onset was inconsistently reported and likely differed within and between studies, which is particularly relevant given that NLR fluctuates across the febrile, critical, and convalescent phases of dengue; comparator definitions differed (for example, “healthy controls” vs. “other febrile illnesses” were not always clearly distinguished); and assay methods for complete blood counts were not standardized across laboratories and countries. Because the same biomarker behaves differently depending on disease phase, admission timing could not be reliably distinguished from later sampling in the severity analyses, which limits the interpretability of these comparisons. Third, additional subgroup analyses by population age (adult vs. paediatric), timing of blood sampling, and diagnostic criteria, as well as meta-regression, were considered but not performed for outcomes beyond DF vs. COVID-19, since most comparisons were informed by a single study (DF vs. healthy controls, malaria, and scrub typhus) or by three studies at most (severity comparisons), well below the minimum number of studies per covariate (typically ≥10) recommended by the Cochrane Handbook to avoid spurious or underpowered findings.

However, our study also has strengths. First, various sensitivity and certainty assessment analyses were performed to ensure both an adequate assessment of heterogeneity and a complete interpretation of our findings. In addition, several comparisons of the usefulness of NLR were performed to evaluate the use of this marker in various scenarios.

## 5 Conclusions

Our review shows that, owing to extreme heterogeneity between studies, variability in cut-off points, and certainty of evidence ranging from moderate to very low, the NLR should be regarded as an adjunctive tool at best rather than a stand-alone diagnostic or prognostic marker for dengue. Given its low cost and ready availability, however, it could have a complementary role in integrated clinical assessment algorithms, particularly in resource-poor settings, if the information it provides can be integrated with other clinical and laboratory parameters. The lack of standardization in the measurement phase, analytical methods and definition of severity also highlights the need for higher quality prospective studies to establish validated cut-off points and make a more accurate assessment of the true operational value of NLR in different epidemiological scenarios.

## Data Availability

The original contributions presented in the study are included in the article/Supplementary Material, further inquiries can be directed to the corresponding author.
