# Supplementary material for: Neutrophil-to-lymphocyte ratio for the clinical management of dengue: a systematic review and meta-analysis
Source: Front Epidemiol. 2026 Aug 26;6:1877646. doi: 10.3389/fepid.2026.1877646 (PMC13553780; doi:10.3389/fepid.2026.1877646)
Supplement: Supplementary file 1 [file Supplementaryfile1.docx]

**Neutrophil-to-Lymphocyte Ratio for the Clinical Management of Dengue: A Systematic Review and Meta-Analysis**

**Supplementary Material S1. Search strategy**

| Database | Search strategy |
| --- | --- |
| Pubmed | #1 Dengue[MH] OR “Breakbone Fev*”[tiab] OR Dengue*[tiab] OR “Break-Bone Fev*”[tiab] OR “Break Bone Fev*”[tiab] OR “Denv virus”[tiab] OR Dengue Virus[MH]  #2 "neutrophil to lymphoc*" [all fields] OR "neutrophil-to-lymphoc*"[all fields] OR "neutrophil/lymphoc*"[all fields] OR "NLR" [all fields] OR "neutrophil lymphoc*" [all fields] OR "granulocyte lymphoc*" [all fields] OR "granulocyte to lymphoc*" [all fields] OR "granulocyteto-lymphoc*"[all fields] OR "granulocyte/lymphoc*" OR "GLR" [all fields]  #3: #1 AND #2 |
| Scopus | #1 TITLE-ABS-KEY(“Breakbone Fev*” OR Dengue* OR “Break-Bone Fev*” OR “Break Bone Fev*” OR “Denv virus”)  #2 TITLE-ABS-KEY("neutrophil to lymphoc*" OR “neutrophil-to-lymphoc*” OR “neutrophil/lymphoc*” OR "NLR" OR "neutrophil lymphoc*" OR "granulocyte lymphoc*" OR “granulocyte to lymphoc*” OR “granulocyteto-lymphoc*” OR “granulocyte/lymphoc*” OR "GLR")  #3: #1 AND #2 |
| WOS | #1 TS= (“Breakbone Fev*” OR Dengue* OR “Break-Bone Fev*” OR “Break Bone Fev*” OR “Denv virus”) OR TI=(“Breakbone Fev*” OR Dengue* OR “Break-Bone Fev*” OR “Break Bone Fev*” OR “Denv virus”) OR AB=(“Breakbone Fev*” OR Dengue* OR “Break-Bone Fev*” OR “Break Bone Fev*” OR “Denv virus”) OR AK=(“Breakbone Fev*” OR Dengue* OR “Break-Bone Fev*” OR “Break Bone Fev*” OR “Denv virus”) OR KP=(“Breakbone Fev*” OR Dengue* OR “Break-Bone Fev*” OR “Break Bone Fev*” OR “Denv virus”)  #2 TS=("neutrophil to lymphoc*" OR “neutrophil-to-lymphoc*” OR “neutrophil/lymphoc*” OR "NLR" OR "neutrophil lymphoc*" OR "granulocyte lymphoc*" OR “granulocyte to lymphoc*” OR “granulocyteto-lymphoc*” OR “granulocyte/lymphoc*” OR "GLR") OR TI=("neutrophil to lymphoc*" OR “neutrophil-to-lymphoc*” OR “neutrophil/lymphoc*” OR "NLR" OR "neutrophil lymphoc*" OR "granulocyte lymphoc*" OR “granulocyte to lymphoc*” OR “granulocytetolymphoc*” OR “granulocyte/lymphoc*” OR "GLR") OR AB=("neutrophil to lymphoc*" OR “neutrophil-to-lymphoc*” OR “neutrophil/lymphoc*” OR "NLR" OR "neutrophil lymphoc*" OR "granulocyte lymphoc*" OR “granulocyte to lymphoc*” OR “granulocyteto-lymphoc*” OR “granulocyte/lymphoc*” OR "GLR") OR AK=("neutrophil to lymphoc*" OR “neutrophil-tolymphoc*” OR “neutrophil/lymphoc*” OR "NLR" OR "neutrophil lymphoc*" OR "granulocyte lymphoc*" OR “granulocyte to lymphoc*” OR “granulocyteto-lymphoc*” OR “granulocyte/lymphoc*” OR "GLR") OR KP=("neutrophil to lymphoc*" OR “neutrophil-tolymphoc*” OR “neutrophil/lymphoc*” OR "NLR" OR "neutrophil lymphoc*" OR "granulocyte lymphoc*" OR “granulocyte to lymphoc*” OR “granulocyteto-lymphoc*” OR “granulocyte/lymphoc*” OR "GLR")  #3: #1 AND #2 |
| Google Scholar | (dengue*) AND ('neutrophil to lymphocyte ratio' OR nlr) |
| Embase | ('dengue'/exp OR 'aden fever' OR 'denv infection' OR 'denv virus infection' OR 'bouquet fever' OR 'break-bone fever' OR 'breakbone fever' OR 'classical dengue' OR 'classical dengue fever' OR 'dandy fever' OR 'dengue' OR 'dengue fever' OR 'dengue virus infection' OR 'infection by denv' OR 'infection by dengue virus' OR 'infection caused by dengue virus' OR 'solar fever' OR 'sun fever' OR 'dengue virus'/exp OR 'denv' OR 'dengue virus' OR 'dengue viruses' OR 'breakbone fever virus' OR 'dandy fever virus' OR 'dengue fever virus' OR 'dengue flavivirus' OR 'denque haemorrhagic virus' OR 'denque hemorrhagic virus') AND ('neutrophil lymphocyte ratio'/exp OR 'nlr (lymphocyte)' OR 'neutrophil lymphocyte ratio' OR 'neutrophil to lymphocyte ratio' OR 'neutrophil/lymphocyte ratio') |

**Supplementary Material S2. Excluded studies**

| **Author** | **Year** | **Tittle** | **Reason for exclusion** |
| --- | --- | --- | --- |
| Rosso | 2021 | Differentiating Dengue from COVID-19: A Diagnostic Challenge in the Tropical Regions of the Americas | Conference abstract |
| Barcelona | 2022 | Complete blood count indices as predictors of severe dengue in pediatric patients admitted at the philippine children's medical center from 2016 to 2019 | Conference abstract |
| Belaunzarán-Zamudio | 2021 | Comparison of clinical characteristics of zika and dengue symptomatic infections and other acute illnesses of unidentified origin in Mexico | Other exposure |
| Kalayanarooj | 1997 | Early Clinical and Laboratory Indicators of Acute Dengue Illness | Other exposure |
| Kain | 2021 | Hematologic Parameters of Acute Dengue Fever Versus Other Febrile Illnesses in Ambulatory Returned Travelers | Conference abstract |
| Priyanka | 2018 | Differentiating between dengue fever from other febrile illnesses using haematological parameters | Other exposure |
| Ralapanawa | 2018 | Value of peripheral blood count for dengue severity prediction | Other exposure |
| Sigera | 2019 | Risk prediction for severe disease and better diagnostic accuracy in early dengue infection; the Colombo dengue study | Other exposure |
| Soh | 2019 | A low lymphocyte: monocyte ratio provides a clue to diagnosing early dengue fever | Other study design |
| Verliyanti | 2023 | Analysis of Platelet Lymphocyte Ratio on Severe COVID-19 and Dengue Hemorrhagic Fever Grade IV | Other exposure |
| Rashmi | 2015 | Haematological and biochemical markers as predictors of dengue infection | Other exposure |
| Naeem | 2022 | Differential Leukocyte and Platelet Counts as Early Diagnostic Indicators in Acute Dengue Infection | Other exposure |
| Mahayanti | 2024 | The relation between the neutrofil lymphocyte ratio (nlr) and the  degree of dengue hemorrhagic fever in the inpatient installation of ari canti hospital for the period of april 2022 – may 2023 | Other outcome |

**Supplementary Material S3**. **PRISMA flowchart of included studies.**

**Identification of studies via databases and registers**

**Identification**

Records identified (n= 193):

PubMed (n=27)
Scopus (n=49)
Web of Science (n=54)
Embase (n=37)
Google Scholar (n=26)

Records removed *before screening*:

Duplicate records removed
(n = 115)

**Screening**

Records screened

(n =78)

Records excluded**

(n = 50)

Reports sought for retrieval

(n = 28)

Reports not retrieved

(n =0)

Reports excluded:
Other exposition = 8
Other study design = 1
Conference abstract = 3

Other outcome = 1

Reports assessed for eligibility

(n = 28)

Studies included = 15

**Included**

**Supplementary Material S4. The Newcastle-Ottawa Scale (NOS) for Assessing the Quality of Nonrandomized Studies in Meta-Analysis**

| **CASE & CONTROL** | **SELECTION** | | | | **COMPARABILITY** | **EXPOSURE** | | |  |  |
| --- | --- | --- | --- | --- | --- | --- | --- | --- | --- | --- |
| **Item** | **Is the case definition adequate?** | **Representativeness of the case** | **Selection of controls** | **Definition of controls** | **Comparability of cases and controls on the basis of design or analysis** | **Asscertainment of exposure** | **Same method of ascertainment for cases and controls** | **Non-Response rate** |  | **Total score (Risk of bias assessment)** |
| Kotepui et al. 2017 | - | * | - | - | ** | * | * | * |  | 6 (Low risk of bias) |
| Tran et al. 2022 | * | * | - | * | ** | * | * | * |  | 8 (Low risk of bias) |

| **COHORT STUDY** | **SELECTION** | | | | **COMPARABILITY** | **EXPOSURE** | | |  |
| --- | --- | --- | --- | --- | --- | --- | --- | --- | --- |
| **Item** | **Representativeness of the exposed cohort** | **Selection of the non exposed cohort** | **Ascertainment of exposure** | **Definition of cohorts** | **Comparability of cohorts on the basis of design or analysis** | **Assessment of outcome** | **Was follow-up long enough for outcomes to occur** | **Adequacy of follow up of cohorts** | **Total score (Risk of bias assessment)** |
| Chatterjee et al. 2020 | * | - | * | * | ** | * | - | - | 6 (Low risk of bias) |
| Recker et al. 2024 | * | * | * | * | ** | * | - | - | 7 (Low risk of bias) |

| **Cross-secional study** | **SELECTION** | | | | COMPARABILITY | EXPOSURE | | | |
| --- | --- | --- | --- | --- | --- | --- | --- | --- | --- |
| **Author** | **Representativeness of the cases** | **Sample size** | **Non-Response rate** | **Ascertainment of the screening/surveillance tool** | **The potential confounders were investigated by subgroup analysis or multivariable analysis** | **Assessment of the outcome** | **Statistical test** | **Total score (Risk of bias assessment)** | |
| Agrawal et al. 2023 | * | * | * | * | - | ** | * | | 7 (Low risk of bias) |
| Ashma et al. 2023 | * | - | * | * | * | ** | * | | 7 (Low risk of bias) |
| Athira et al. 2018 | * | - | * | * | - | ** | * | | 6 (Low risk of bias) |
| Bansal et al. 2021 | * | - | * | * | - | ** | * | | 6 (Low risk of bias) |
| Bhattarai et al. 2023 | * | - | * | * | * | ** | * | | 7 (Low risk of bias) |
| Hasanah et al. 2023 | * | * | * | * | - | ** | * | | 7 (Low risk of bias) |
| Juliansen et al. 2022 | * | * | * | * | - | ** | * | | 7 (Low risk of bias) |
| Navya et al. 2024 | * | - | * | * | - | ** | * | | 6 (Low risk of bias) |
| Osuna-Ramos et al. 2022 | * | - | * | * | * | ** | * | | 7 (Low risk of bias) |
| Rosso et al. 2020 | * | - | * | * | * | ** | * | | 7 (Low risk of bias) |
| Rosso et al. 2021 | * | - | * | * | * | ** | * | | 7 (Low risk of bias) |

**Supplementary Material S5**. Mean difference in the Neutrophil to Lymphocyte Ratio (NLR) between patients with dengue fever (DF) and fever of unknown etiology according to the exclusions of each study.

**Supplementary Material S6**. Mean difference in the Neutrophil to Lymphocyte Ratio (NLR) between patients with dengue fever (DF) and COVID-19, grouped by geographic region.

**Supplementary Material S7**. Mean difference in the Neutrophil to Lymphocyte Ratio (NLR) between patients with dengue fever (DF) and COVID-19 according to the exclusions of each study.

**Supplementary Material S8**. Mean difference in the Neutrophil to Lymphocyte Ratio (NLR) between patients with dengue fever (DF) with and without warning signs.

**Supplementary Material S9**. Mean difference in the Neutrophil to Lymphocyte Ratio (NLR) between patients with dengue fever (DF) with and without warning sign according to the exclusions of each study.

**Supplementary Material S10.** Mean difference in the Neutrophil to Lymphocyte Ratio (NLR) between patients with severe dengue fever (DF) and DF fever with warning signs.

**Supplementary Material S11.** Mean difference in the Neutrophil to Lymphocyte Ratio (NLR) between patients with severe dengue fever (DF) and DF with warning signs according to the exclusions of each study.

**Supplementary Material S12.** Mean difference in the Neutrophil to Lymphocyte Ratio (NLR) between patients with severe dengue ferver (DF) and DF without warning signs.

**Supplementary Material S13.** Mean difference in the Neutrophil to Lymphocyte Ratio (NLR) between patients with severe dengue fever (DF) and DF without warning signs according to the exclusions of each study.
